# Repurposing Pimozide as a Geroprotector: Lifespan Extension via SKN-1 Activation and Collagen Remodeling in *Caenorhabditis elegans*

**DOI:** 10.3390/antiox15091217

**Published:** 2026-09-21

**Authors:** Yuhong Huang, Meijing Wang, Yiming Zhou, Jiali Liu, Lili Ni, Ya Tan, Mengting Zhang, Jiangpeng Huang, Guolin Li, Fang Wei

**Affiliations:** 1 Center for Aging Biomedicine, National & Local Joint Engineering Laboratory of Animal Peptide Drug Development, College of Life Sciences, Hunan Normal University, 36 Lushan Road, Changsha 410081, China; 2 Key Laboratory of Hunan Province for Model Animal and Stem Cell Biology, School of Medicine, Hunan Normal University, 371 Tongzipo Road, Changsha 410013, China

**Keywords:** pimozide, aging, stress resistance, *skn-1*/Nrf2, collagen, extracellular matrix

## Abstract

Aging is a major risk factor for chronic diseases, driving an urgent need for effective geroprotectors. Repurposing psychotropic agents offers an efficient strategy to accelerate anti-aging therapeutics development. In this study, we investigated the effects of pimozide (a diphenylbutylpiperidine-class antipsychotic) on aging in *Caenorhabditis elegans* and elucidated the underlying mechanisms. The results demonstrated that 0.1 μM pimozide significantly extends nematode lifespan, increasing mean, median, and maximum lifespans by 44.3%, 50.0%, and 39.8%, respectively. Furthermore, pimozide improves healthspan, as evidenced by reduced age-related lipofuscin accumulation, preserved locomotor function, and decreased paralysis incidence. Moreover, pimozide-treated nematodes showed enhanced resistance to heat, ultraviolet and oxidative stress, concomitant with reduced endogenous reactive oxygen species levels. Transcriptomic profiling and biochemical validation reveal that pimozide markedly upregulates collagen gene expression and increases hydroxyproline content, indicating enhanced collagen synthesis. Mechanistically, pimozide promotes the nuclear localization of SKN-1, and its beneficial effects on lifespan extension and collagen synthesis are SKN-1-dependent, as these effects are abolished in *skn-1* mutants. Collectively, our findings demonstrate that pimozide promotes longevity and healthspan by activating *skn-1*, which is accompanied by enhanced collagen synthesis, providing a preclinical proof of concept for its potential repurposing as a geroprotector.

## 1. Introduction

Aging represents a progressive physiological decline and constitutes a primary risk factor for various chronic diseases, including cancers, neurodegenerative disorders, and cardiovascular diseases [1]. An aging population puts increased pressure on healthcare systems. Consequently, there is an urgent need to identify intervention strategies capable of delaying the incidence of age-associated pathologies and prolonging healthspan [2]. However, the *de novo* development of anti-aging therapeutics encounters substantial bottlenecks, such as prolonged development timelines, exorbitant costs, and high clinical translation failure rates; to date, no geroprotector has been approved for clinical use [1,3]. In this milieu, drug repurposing can accelerate early-stage development, thereby emerging as an efficient avenue for geroprotector discovery [4]; nevertheless, clinical translation remains challenging, requiring rigorous validation of safety, dosage, and efficacy for new indications.

Among various candidate therapeutics, certain psychotropic medications have demonstrated anti-aging potential. These agents target monoamine neurotransmitter systems, glutamatergic signaling, redox signaling, or epigenetic regulators to modulate core aging-associated processes, including autophagy, mitochondrial homeostasis, inflammation, and oxidative stress [5]. For instance, the monoamine oxidase B (MAO-B) inhibitor selegiline shows consistent effects on lifespan expectancy across multiple animal models [6]. Recently, the antidepressant mianserin and specific serotonin reuptake inhibitors (SSRIs) have garnered attention, demonstrating the capacity to extend both lifespan and healthspan of *Caenorhabditis elegans* (*C. elegans*) [7,8,9,10,11]. Importantly, the geroprotective efficacy of psychotropic drugs is not universal, as their effects are dependent on the specific compound and dosage. For example, within the second-generation antipsychotics, aripiprazole extends lifespan, whereas quetiapine shortens it [12]. Furthermore, several SSRIs prolong lifespan only at lower doses and lose efficacy at higher concentrations [7,9,10,11].

Pimozide, a first-generation diphenylbutylpiperidine antipsychotic, is widely used clinically to treat conditions such as schizophrenia, delusional infestation and Tourette syndrome [13]. Mechanistically, it functions not only as a dopamine D2 receptor (D2R) antagonist and calcium channel blocker, but also as an antagonist of 5-HT2 and 5-HT7 receptors [13,14]. Pimozide exhibits considerable potential against aging-related neurodegeneration. Its distinctive benzimidazole structure endows it with superior synergistic anti-Alzheimer’s disease potential compared to other antipsychotics within the same class. Moreover, it exerts multifaceted neuroprotective effects, including the inhibition of β-amyloid aggregation and protection of astrocytes against oxidative damage [15]. Furthermore, pimozide alleviates motor neurodegeneration and preserves muscle strength across diverse models by stabilizing and restoring neuromuscular transmission [16]. Beyond these neuroprotective properties, emerging evidence indicates that pimozide can induce autophagy, elicit anti-inflammatory effects, and regulate several key aging-associated signaling pathways [17,18], all of which are central to the regulation of organismal aging. However, these findings are largely restricted to *in vitro* systems or specific disease models, and no study has assessed its direct impact on natural aging and healthspan at the whole-organism level.

*C. elegans* is widely recognized as an optimal model for anti-aging drug screening and mechanistic research, owing to its short lifespan, well-characterized genetic background, and highly conserved aging pathways [19]. Accordingly, this study aims to evaluate the effects of pimozide on lifespan and healthspan in *C. elegans* and to elucidate its potential molecular mechanisms via transcriptomic analysis. This work seeks to provide data supporting pimozide repurposing and to establish a theoretical foundation for psychotropic-based anti-aging intervention strategies.

## 2. Materials and Methods

### 2.1. Chemicals and Reagents

Pimozide was obtained from Aladdin (P275736, Shanghai, China). Juglone was obtained from Bidepharm (BD12556, Shanghai, China). 2,7-dichlorodihydrofluorescein diacetate (DCFH-DA) was purchased from Beyotime (S1105, Shanghai, China). Dimethyl sulfoxide (DMSO) was acquired from Sangon Biotech (A600163, Shanghai, China). Ampicillin sodium (IA0340) and other routine chemicals for *C. elegans* experiments were purchased from Solarbio (Beijing, China). Levamisole hydrochloride was obtained from Abmole (M5348, Houston, TX, USA).

### 2.2. C. elegans Strains

Wild-type N2, LD1 [*skn-1*b/c::GFP + *rol-6*(su1006)] and EU-1 [*skn-1*(*zu67*)] were utilized in this study, with the latter kindly provided by Y Liang from Central South University of Forestry & Technology (Changsha, China). *skn-1*(*zu67*) homozygotes were identified by their non-Unc phenotype among progeny from heterozygous parents. Unless otherwise specified, all nematodes were cultured on nematode growth medium (NGM) at 20 °C, utilizing *Escherichia coli* (*E. coli*) OP50 as the standard food source. To obtain age-synchronized populations, gravid hermaphrodites were subjected to a standard hypochlorite bleaching procedure (A501944, Sangon, Shanghai, China).

### 2.3. Pharmacological Intervention

Pimozide was dissolved in DMSO to prepare stock solutions, which were diluted into molten NGM agar to achieve the indicated working concentrations with a final DMSO concentration of 0.1% (*v*/*v*) in all plates, including vehicle controls. For the thermotolerance assay, pimozide was administered at concentrations of 0.1, 0.2, 0.5, 1, 10 and 30 μM. A concentration of 0.1 μM was used in all other experiments. Age-synchronized worms at the L4 larval stage were randomly assigned to the vehicle control or pimozide group. The initiation of treatment was designated as day 0. To ensure continuous drug exposure and a fresh food supply, worms were relocated to freshly prepared treatment plates every 2 days.

### 2.4. Lifespan Assay

Worms were monitored daily for mortality. Survival was assessed by evaluating the presence or absence of a physiological response to mechanical stimulation with a platinum wire. Worms that died of bagging, vulval rupture, internal hatching, or crawling off the agar were censored. A survival curve was generated using the Kaplan–Meier method, and statistical differences were determined by the log-rank test. Three independent biological replicates were performed, with 50–55 worms per group in each assay.

### 2.5. Motility Assay

Locomotor activity was assessed on days 6 and 12. Head swing frequency was manually counted over 1-min intervals. Furthermore, overall movement was visually categorized into three grades: Grade A, spontaneous sinusoidal movement; Grade B, stimulus-induced non-sinusoidal movement; and Grade C, immobility even after stimulus. Three independent biological replicates were performed, with 30 worms per group in each assay.

### 2.6. Analysis of Lipofuscin Accumulation

Worms were paralyzed using 5 µM levamisole and mounted onto 1% agarose pads. Imaging of intestinal lipofuscin was conducted utilizing an Olympus IX71 inverted fluorescence microscope (Tokyo, Japan). Whole-field imaging was performed without pre-selection of specific regions of interest; all worms within the field of view were captured. Subsequent quantification of fluorescence signals was performed with Image-Pro Plus software (version 7.1, Media Cybernetics, MD, USA). Three independent biological replicates were performed, with 8 worms per group in each assay. Investigators were blinded to group allocation throughout the experiment.

### 2.7. Measurement of Reactive Oxygen Species (ROS)

Worms were washed three times with M9 buffer. Intracellular ROS levels were detected using the fluorescent probe DCFH-DA, with imaging and quantification performed following the same protocols as described for lipofuscin detection, including whole-field imaging without pre-selection of specific regions of interest. Three independent biological replicates were performed, with 8 worms per group in each assay, and investigators were blinded to group allocation.

### 2.8. Fertility Assay

Synchronized L4-stage worms per group were subjected to the designated treatments (Day 0). Parental worms were transferred to fresh plates every 24 h until egg-laying ceased. The eggs were then incubated, and the progeny were counted under a microscope. Three independent biological replicates were performed.

### 2.9. Bacterial Growth Assay

Overnight cultures of OP50 were diluted to an OD_600_ of 0.1 and treated with either pimozide or vehicle control. Bacterial growth was monitored by measuring OD_600_ at 3, 6, 9, 12, and 24 h of incubation. Three independent biological replicates were performed.

### 2.10. Stress Resistance Assays

Worms were subjected to various lethal stressors. The death criteria and survival analysis followed the same protocols established for the lifespan assay. For the heat stress assay, worms were incubated at 36.5 °C (hour 0), and mortality was recorded hourly. For the ultraviolet (UV) irradiation assay, worms were subjected to a single dose of 120 mJ/cm^2^ UV radiation (day 0), and survival was monitored daily. For the oxidative stress assay, worms were transferred to NGM plates containing 500 μM juglone (hour 0), and mortality was recorded hourly. Three independent biological replicates were performed, with 50–55 worms per group in each assay.

### 2.11. Hydroxyproline Estimation

The total hydroxyproline content was measured as an established biochemical marker for collagen accumulation [20]. Worms were collected and washed with M9 buffer, and the lysates were then analyzed using a colorimetric hydroxyproline assay kit (Abbkine, Wuhan, China) in strict accordance with the manufacturer’s instructions. Three independent biological replicates were performed, with at least 2000 worms per group in each assay.

### 2.12. SKN-1 Nuclear Localization Assay

The LD1 strain was used to assess SKN-1 nuclear localization. The procedures for worm preparation and image acquisition were identical to those described for the lipofuscin accumulation assay. As described previously [21], animals were categorized into low, medium, or high nuclear localization groups based on the distribution and intensity of intestinal SKN-1B/C::GFP signals. Briefly, low accumulation represented a barely visible signal; medium indicated a strong SKN-1B/C::GFP signal in the anterior and/or posterior intestinal nuclei while being faint in the mid-intestinal region, or that a weak signal was observed throughout all intestinal nuclei; and high denoted a strong signal distributed throughout all intestinal nuclei. Investigators responsible for image acquisition and nuclear-localization scoring were blinded to group allocation. Three independent biological replicates were performed, with 30 worms per group in each assay.

### 2.13. RNA Extraction, cDNA Synthesis, and Real-Time PCR

Nematodes exposed to pimozide or vehicle control were subjected to total RNA isolation via TRIzol reagent (real-time PCR: 5 biological replicates, with 300 worms per group; RNA sequencing: 4 biological replicates, with 2000 worms per group). Synthesis of cDNA from 200 ng of RNA templates was carried out using the cDNA Synthesis Mix (Takara, Dalian, China). qPCR assays were executed on an Applied Biosystems QuantStudio 5 platform utilizing SYBR Green chemistry (Novoprotein, Suzhou, China) and gene-specific primers (sequences listed in Table S1). The thermocycling conditions were: 95 °C for 30 s, then 95 °C for 5 s, and 60 °C for 15 s in 40 cycles. Melting curve analysis confirmed amplicon specificity. Relative gene expression was quantified via the 2^−ΔΔCT^ approach. *act-1* was chosen as the normalization control, as its expression remained stable across all experimental samples, and this choice is supported by a previous study [22]. Potential outliers were initially identified using box-and-whisker plots and subsequently evaluated using Grubbs’ test [23].

### 2.14. Library Generation and Sequencing

As described previously [24], poly(A)-enriched mRNA was captured from total RNA through two rounds of Dynabeads Oligo(dT)25 purification (Thermo Fisher, Waltham, MA, USA) and then fragmented with the Magnesium RNA Fragmentation Module (NEB, Ipswich, MA, USA) at 94 °C for 5–7 min. First-strand cDNA was generated using SuperScript^TM^ II Reverse Transcriptase (Invitrogen, Carlsbad, CA, USA), followed by second-strand synthesis using *E. coli* DNA polymerase I (NEB, Ipswich, MA, USA), RNase H (NEB, Ipswich, MA, USA), and dUTP incorporation (Thermo Fisher, Waltham, MA, USA). After end repair, A-tailing and adapter ligation, size selection was performed using AMPure XP beads. The dUTP-containing second strand was selectively degraded by Uracil-DNA-Glycosylase (NEB, Ipswich, MA, USA). The remaining strand was PCR-amplified (95 °C for 3 min, 8 cycles of “98 °C for 15 s, 60 °C for 15 s, and 72 °C for 30 s”, and 72 °C for 5 min) to generate a cDNA library with 300 ± 50 bp. The libraries were pooled in equimolar concentrations and sequenced on an Illumina Novaseq^TM^ 6000 platform using a 2 × 150 bp paired-end strategy (LC-Bio Technology Co., Ltd., Hangzhou, China). Raw sequence data have been deposited in the GEO database under accession number GSE338120 and are publicly available at: https://www.ncbi.nlm.nih.gov/geo/query/acc.cgi?acc=GSE338120 (accessed on 19 August 2026).

### 2.15. Data Processing

Raw sequencing reads were processed with Cutadapt to remove adapter sequences and low-quality reads [25]. All samples had Q30 values above 90%. High-quality reads were aligned to the *C. elegans* reference genome (WBcel235) using the HISAT2 aligner [26], with mapping rates exceeding 95% (Table S2). Transcripts were assembled using StringTie with default parameters [27], and the resulting FPKM values were imported into Ballgown [27,28]. Gene-level raw counts were analyzed using DESeq2 with its default independent-filtering procedure for low-abundance genes [29]. Genes with a Benjamini–Hochberg adjusted *p* value (padj) < 0.05 and |log2 fold change| > 1 were considered differentially expressed.

For principal component analysis (PCA), raw counts were variance-stabilized using DESeq2. The 500 genes with the highest variance across samples were selected and analyzed using the prcomp function in R, and the first two principal components were visualized using ggplot2. GO and KEGG enrichment analyses were performed separately for the upregulated and downregulated genes. GO analysis was conducted using the enrichGO function in clusterProfiler (version 4.20.0) with org.Ce.eg.db (version 3.22.0), whereas KEGG analysis was performed using the enricher function with *C. elegans* pathway annotations retrieved through KEGGREST [30,31,32]. All genes that passed DESeq2 independent filtering and were represented in the corresponding annotation database were used as the background. Enrichment was evaluated using a hypergeometric test, followed by Benjamini–Hochberg correction. Terms or pathways with an adjusted *p* value < 0.05 were considered significantly enriched.

### 2.16. Statistical Analysis

Data were assessed for normality and homogeneity of variance before parametric testing. Two-group comparisons were analyzed by unpaired Student’s *t*-test, and multiple-group comparisons were analyzed by one-way analysis of variance (ANOVA) followed by Tukey’s post-hoc test. Data involving genotype and drug treatment, and OP50 growth data across time and treatment, were analyzed using two-way ANOVA followed by Tukey’s and Šídák’s post-hoc tests, respectively. Survival curves were compared using the log-rank (Mantel–Cox) test. Hazard ratios (HRs) and their 95% confidence intervals (CIs) were calculated using the Cox proportional hazards model. Statistical analyses were carried out in GraphPad Prism 9.0 or SPSS 17, with *p* < 0.05 considered statistically significant.

## 3. Results

### 3.1. Pimozide Prolongs the Lifespan of C. elegans

**Figure 1:**
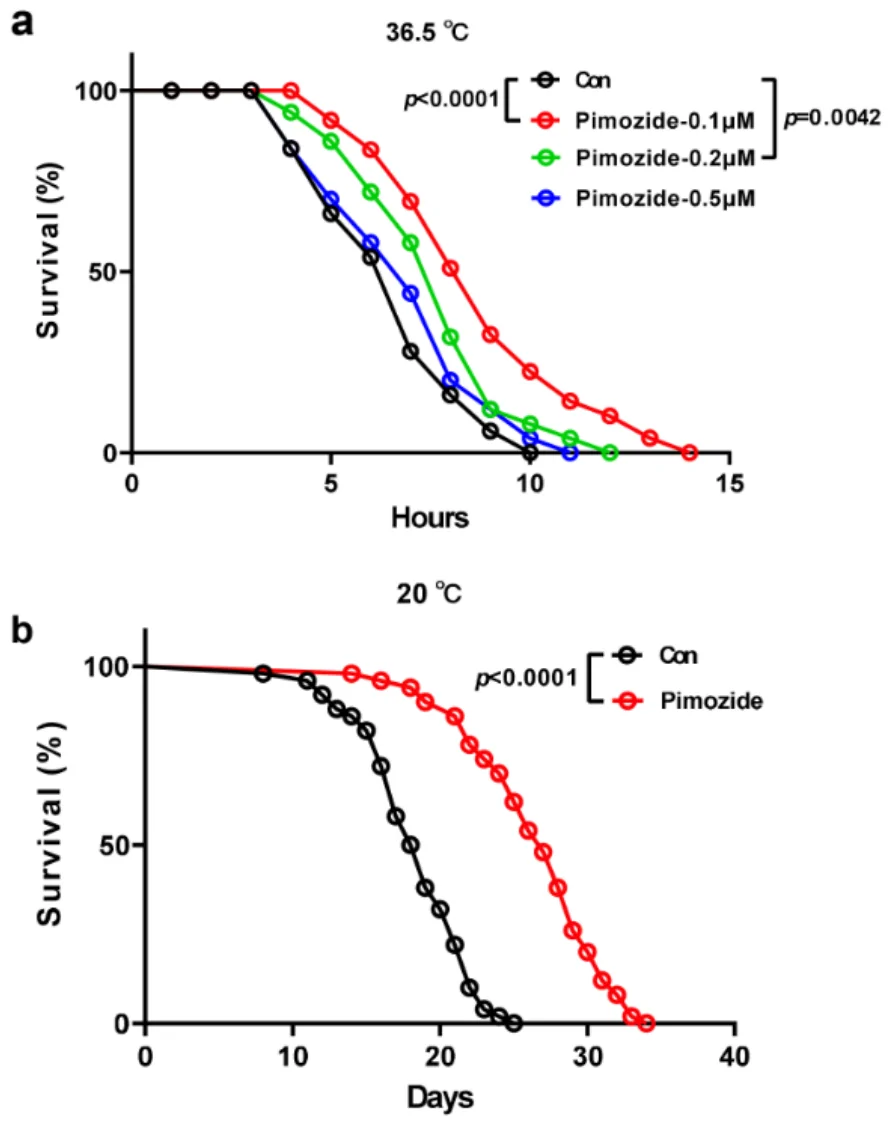
Pimozide prolongs the lifespan of *C. elegans*. Survival curves for nematodes maintained on NGM plates containing either vehicle (Con, 0.1% DMSO) or pimozide under 36.5 °C (**a**) or 20 °C (**b**). Each concentration of pimozide was compared with the vehicle control, and the *p* values are shown beside the corresponding brackets. Replicates are shown in Figure S1. Sample sizes and log-rank *p*-values are summarized in Table S3. Con, control; DMSO, dimethyl sulfoxide; NGM, nematode growth medium.

Because lifespan extension is frequently linked to enhanced stress resistance [33], we initially conducted a rapid preliminary screen under heat stress (36.5 °C) to evaluate the impact of pimozide on thermotolerance. Previous studies have shown that 40 μM pimozide ameliorates paralysis and motor-neuron degeneration in a mutant TDP-43 *C. elegans* model [16]. On this basis, we first conducted a broad-range concentration screen (0.1, 1, 10, and 30 μM) to assess pimozide effects on heat stress resistance. Among the concentrations tested, only 0.1 μM pimozide significantly prolonged survival, whereas higher concentrations (1, 10, and 30 μM) showed no significant effect (Figure S1a). To further characterize the dose–response relationship within this low concentration window, we subsequently performed heat stress survival assays using 0.1, 0.2, and 0.5 μM pimozide. Both 0.1 and 0.2 μM significantly prolonged survival during heat stress, whereas 0.5 μM did not. The most robust lifespan extension was observed at 0.1 μM. Under heat stress, control worms exhibited a mean, median, and maximum lifespan of 6.5, 7.0, and 9.6 h, respectively. Treatment with 0.1 μM pimozide extended these values to 8.8, 9.0, and 13.5 h, representing increases of approximately 35.4%, 28.6%, and 40.6%, respectively (Figure 1a and Figure S1b,c; Table S3; percentages calculated from representative data in Figure 1a). To corroborate these findings, we monitored lifespan under standard culture conditions (20 °C). Consistent with the heat stress data, 0.1 μM pimozide significantly promoted longevity under normal conditions, with control worms surviving a mean, median, and maximum of 18.3, 18.0, and 23.6 days compared to 26.4, 27.0, and 33.0 days in the treated group, corresponding to increases of approximately 44.3%, 50.0%, and 39.8%, respectively (Figure 1b and Figure S1d,e; Table S3; percentages calculated from representative data in Figure 1b). Collectively, these data demonstrate that pimozide enhances stress resistance and promotes longevity in *C. elegans* with optimal efficacy at 0.1 µM; therefore, this dosage was selected for all subsequent experiments.

### 3.2. Pimozide Enhances Healthspan in C. elegans

**Figure 2:**
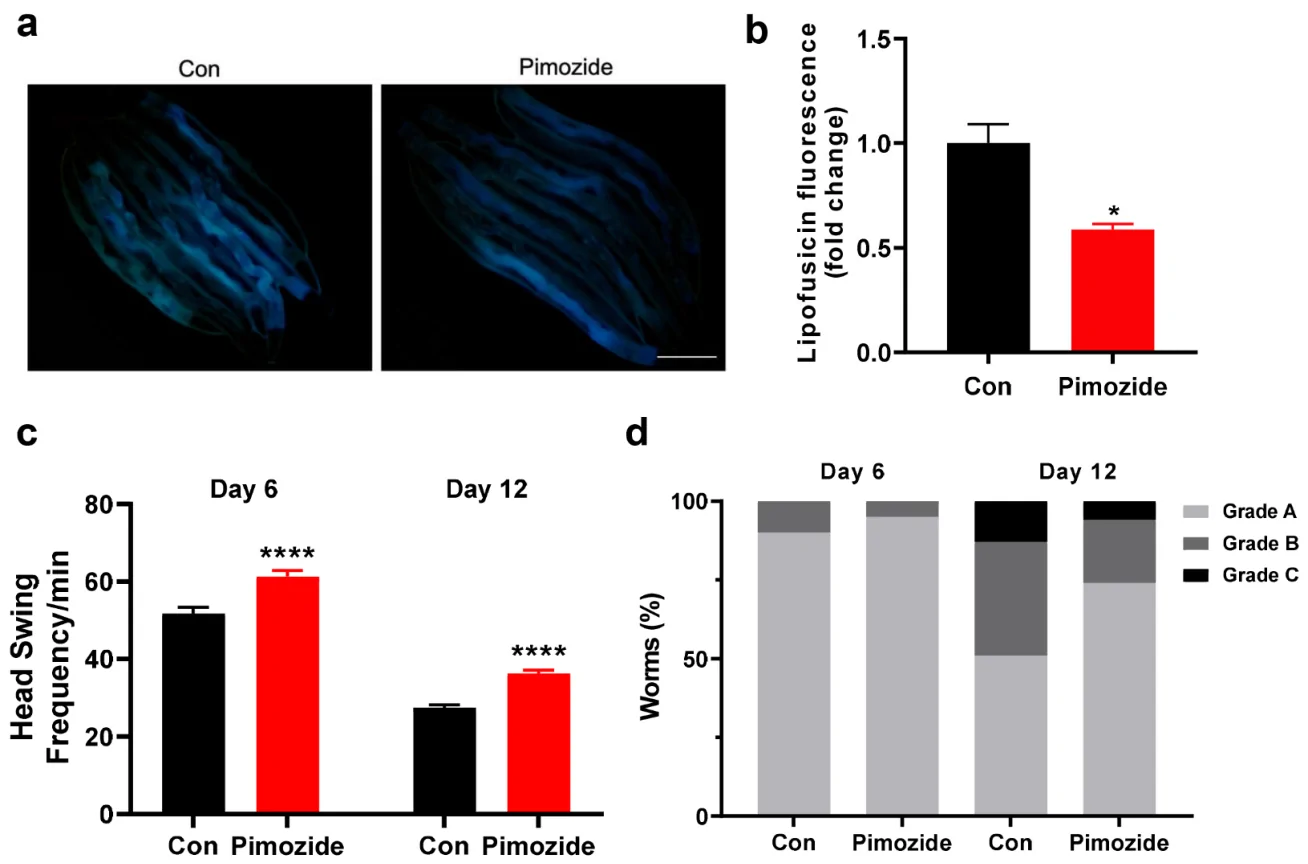
Pimozide prolongs healthspan of *C. elegans*. (**a**,**b**) Representative fluorescent image (**a**) and quantification (**b**) of intestinal lipofuscin accumulation in vehicle- or pimozide-treated worms. Scale bar: 0.5 mm, *n* = 8. (**c**,**d**) Effects of pimozide on locomotor performance at days 6 and 12, measured as head-swing frequency per minute (**c**) and body-bend scoring (**d**). Grade A: spontaneous sinusoidal movement. Grade B: stimulus-induced non-sinusoidal movement. Grade C: immobility even after stimulus. Data are mean ± SEM. Asterisks indicate statistical significance in pimozide versus Con (*, *p* < 0.05; ****, *p* < 0.0001). Error bars: SEM. Con: control.

To investigate whether pimozide-mediated lifespan extension is accompanied by improved healthspan, we evaluated nematode motility and the accumulation of lipofuscin, a well-established aging biomarker. Compared to controls, pimozide-treated nematodes exhibited a significant reduction in lipofuscin fluorescence intensity (Figure 2a,b). Furthermore, the age-related decline in head-swing frequency was significantly mitigated by pimozide, preserving this locomotor function in both mature (6 days) and aged (12 days) nematodes (Figure 2c). Consistent with these findings, pimozide increased the proportion of aged nematodes maintaining autonomous sinusoidal movement and reduced the incidence of paralysis (Figure 2d). Additionally, pimozide did not affect OP50 growth and reproductive capacity of worms (Figure S2a,b). In summary, these results indicate that pimozide extends lifespan and concurrently improves late-life physiological function and overall healthspan in *C. elegans*.

### 3.3. Pimozide Enhances Stress Resistance in C. elegans

**Figure 3:**
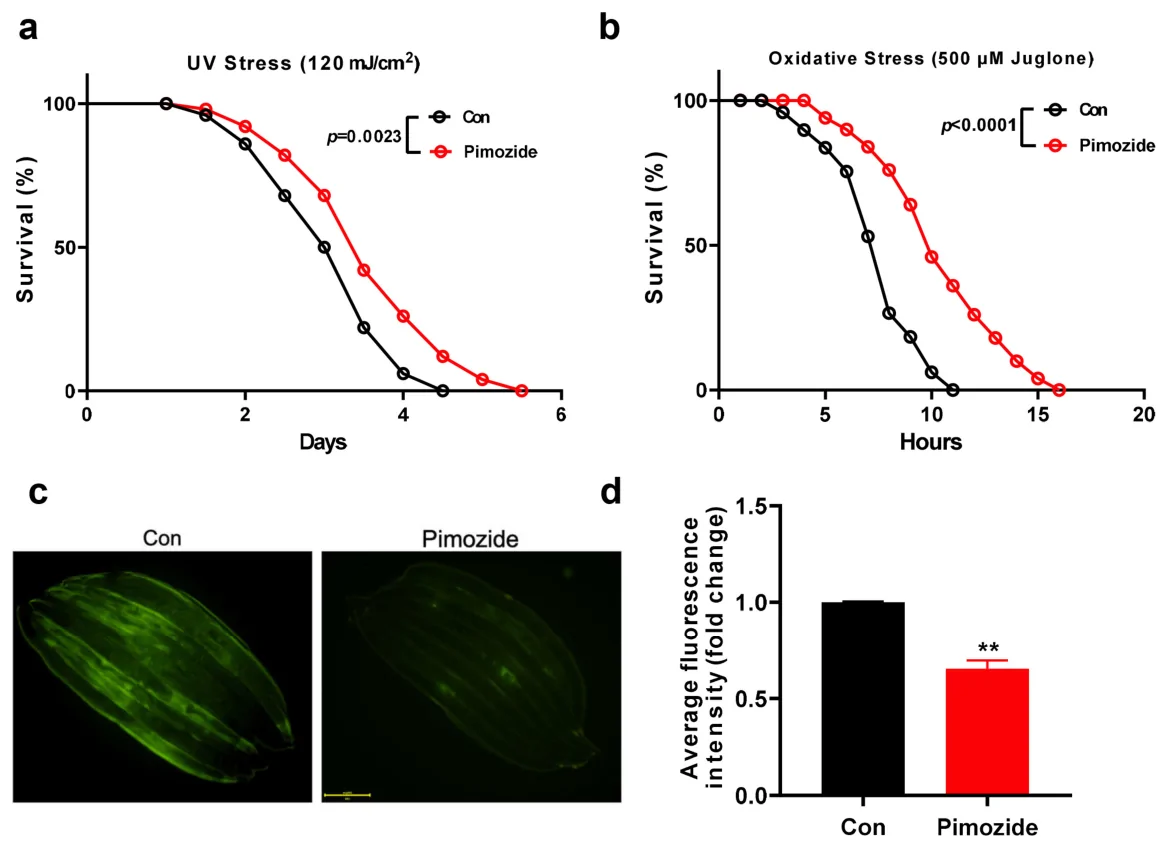
Pimozide improves stress tolerance in *C. elegans*. (**a**,**b**) Representative survival curves show vehicle- or pimozide-treated worms challenged with UV irradiation at 120 mJ/cm^2^ (**a**) or 500 μM juglone (**b**) until all the worms died. Replicates are shown in Figure S2. Sample sizes and log-rank *p*-values are summarized in Table S4. (**c**,**d**) ROS fluorescent micrograph (**c**) and quantification (**d**) of vehicle- or pimozide-treated worms. Scale bar: 0.2 mm, *n* = 8. Data are mean ± SEM. Asterisks indicate statistical significance in pimozide versus Con (**, *p* < 0.01). Error bars: SEM. Con: control. ROS: reactive oxygen species. UV: ultraviolet.

As demonstrated above, pimozide enhanced heat stress resistance in *C. elegans*, and given that stress resistance is a conserved hallmark of longevity interventions [34], we further evaluated its protective effects against UV irradiation and oxidative stress. Relative to controls, pimozide-treated nematodes displayed significantly extended survival under both stressors. Under UV stress, control worms exhibited mean, median, and maximum survival times of 3.1, 3.0, and 4.3 days, respectively, whereas pimozide-treated worms showed 3.6, 3.5, and 5.2 days, corresponding to increases of approximately 16.1%, 16.7%, and 20.9% (Figure 3a and Figure S2c,d; Table S4; percentages calculated from representative data in Figure 3a). Under oxidative stress, the mean, median, and maximum survival times were 7.5, 8.0, and 10.6 h for controls and 10.5, 10.0, and 15.4 h for pimozide-treated worms, representing increases of 40.0%, 25.0%, and 45.3%, respectively (Figure 3b and Figure S2e,f; Table S4; percentages calculated from representative data in Figure 3b). Furthermore, we observed a significant reduction in endogenous ROS levels following pimozide treatment, providing a mechanistic basis for their resilience to oxidative stress. These results indicate that pimozide fortifies *C. elegans* against diverse environmental stressors.

### 3.4. Pimozide Promotes Collagen Synthesis in C. elegans

**Figure 4:**
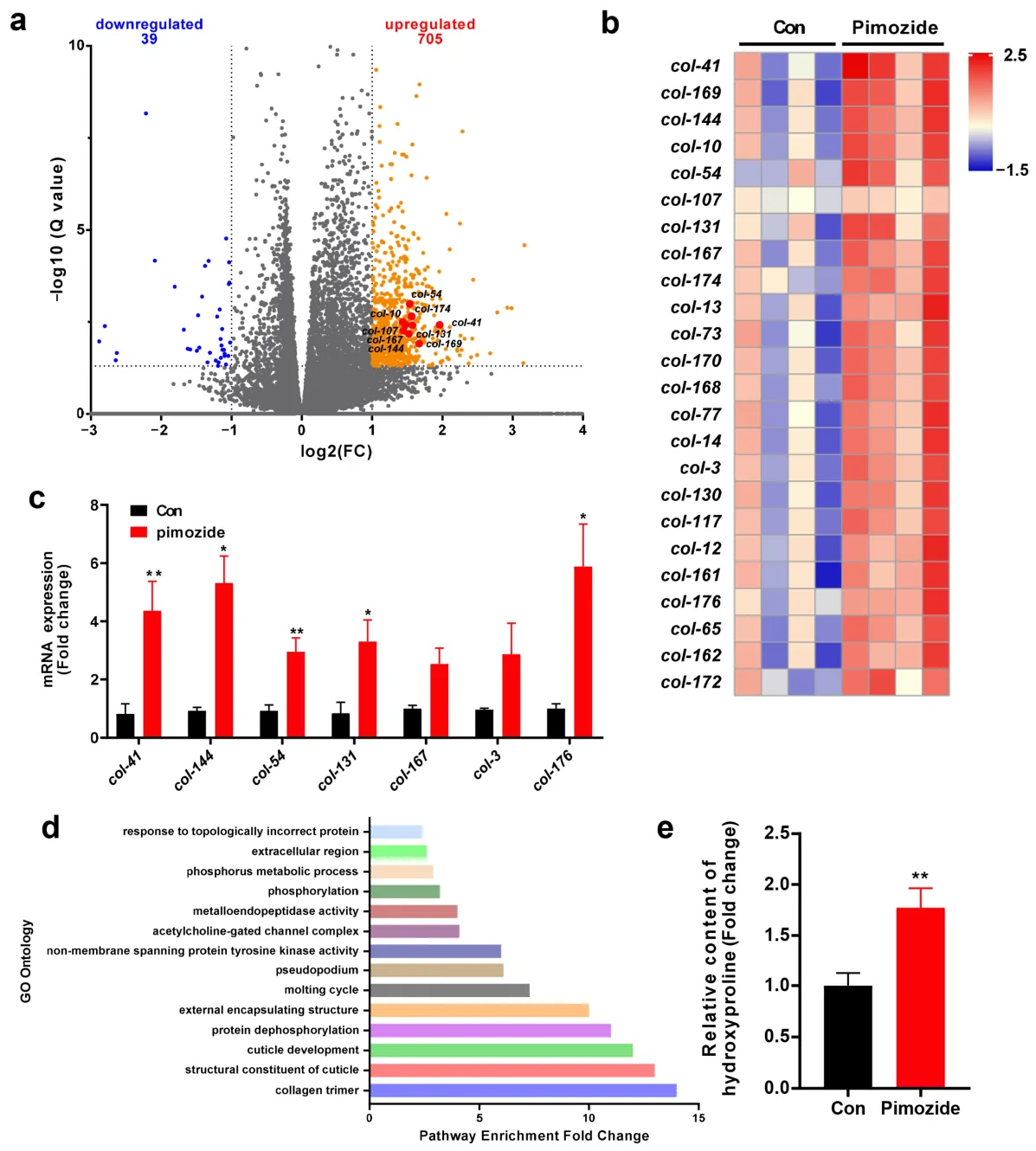
Pimozide promotes collagen remodeling in *C. elegans*. (**a**) Differential expression profile visualized by volcano plot: orange, upregulated; blue, downregulated; gray, no statistically significant difference; red, upregulated collagen genes. (**b**) Heat maps showing differentially expressed collagen genes regulated by pimozide; cells are shaded according to log_2_(fold change to control) from −1.5 to 2.5 (blue for low, red for high). (**c**) The effect of pimozide on mRNA expression (fold change relative to control worms) of collagen genes. (**d**) Gene ontology enrichment analysis of pimozide-induced DEGs. (**e**) Total collagen is indicated by hydroxyproline content. Data are mean ± SEM. Asterisks indicate statistical significance in the corresponding pimozide versus Con (*, *p* < 0.05; **, *p* < 0.01). Error bars: SEM. Con: control. FC: fold change.

To elucidate the molecular mechanism underlying pimozide-induced longevity, we performed transcriptomic profiling of pimozide-treated and control nematodes. The analysis revealed 744 differentially expressed genes (DEGs), with 705 upregulated and 39 downregulated (Figure 4a). Notably, 25 of the upregulated genes belonged to the collagen family, with the top candidates exhibiting fold changes exceeding 3, several of which were subsequently validated by qRT-PCR (Figure 4b,c). PCA showed clear separation between the two groups along PC1, with PC1 and PC2 explaining 81.2% and 9.9% of the variance, respectively, confirming high reproducibility and indicating that treatment-related transcriptional differences dominate sample variation (Figure S3a). KEGG enrichment analysis revealed that upregulated DEGs were significantly enriched in the integrated stress response signaling pathway, mRNA surveillance pathway, and cadherin signaling, whereas no significant pathways were enriched among downregulated DEGs (Figure S3b). GO enrichment analysis indicated that the upregulated DEGs were highly enriched for terms including “collagen trimer”, “structural constituent of cuticle”, and “cuticle development” (Figure 4d). In contrast, the 39 downregulated genes were primarily enriched in overlapping GO terms related to responses to biotic stimuli and innate immunity, driven mainly by *B0024.4*, *fbxc-58*, *F35E8.10*, *C49G7.13*, *lys-3*, and *ilys-3* (Figure S3c). Given the limited number and narrow scope of these genes, pimozide may exert moderate transcriptional suppression on specific immune-related modules rather than broad immunosuppression. As the major structural component of the *C. elegans* cuticle, collagen is essential for maintaining body integrity and has been linked to lifespan regulation [35,36]. We therefore hypothesized that pimozide may promote longevity by stimulating collagen synthesis. Supporting this premise, pimozide treatment significantly increased hydroxyproline levels, a characteristic biochemical marker of collagen content (Figure 4e). Collectively, these results suggest that pimozide extends lifespan while simultaneously promoting collagen synthesis and maintaining cuticular structural homeostasis.

### 3.5. Pimozide Activates SKN-1 to Extend C. elegans Lifespan and Promote Collagen Synthesis

**Figure 5:**
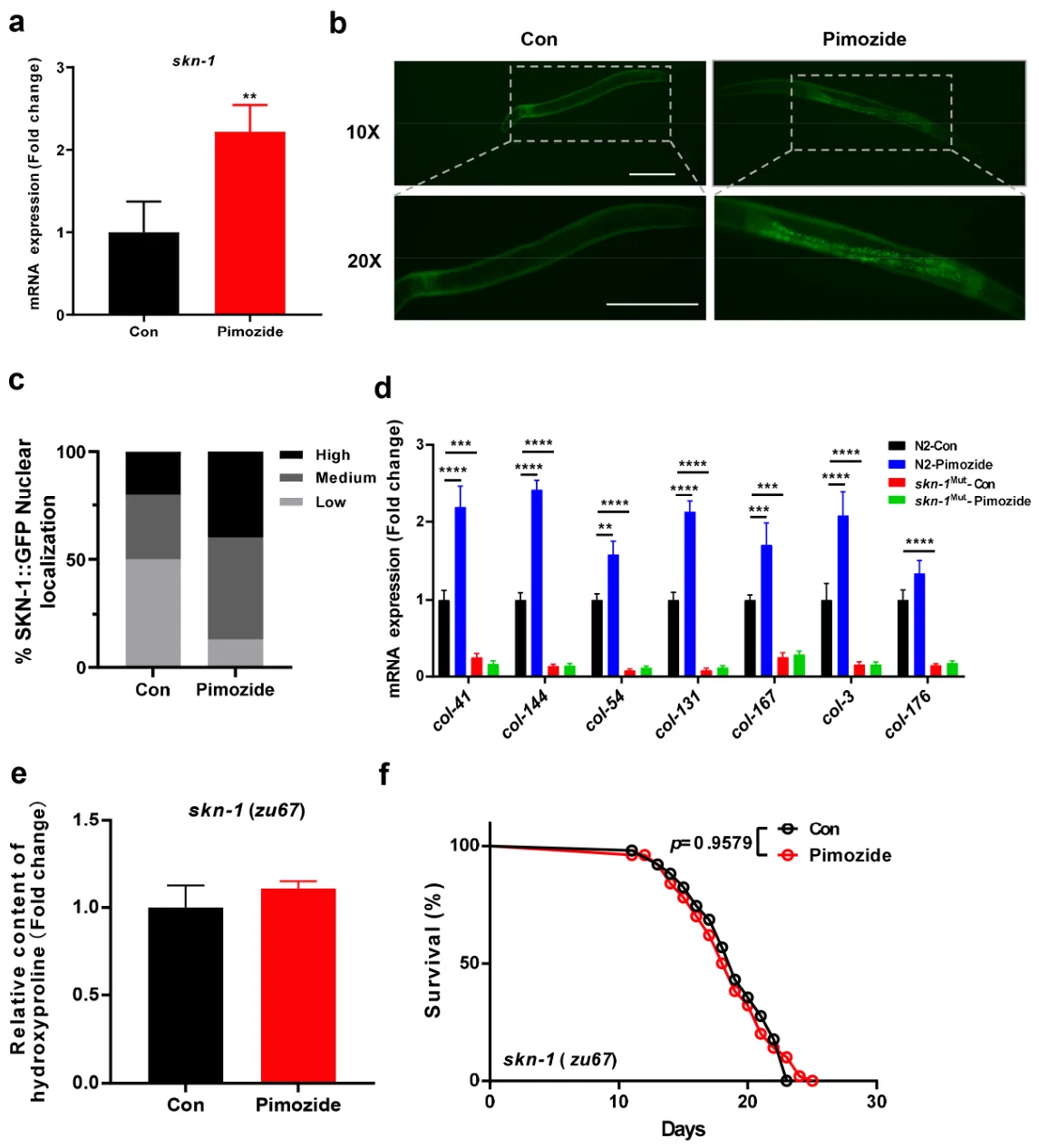
Pimozide activates SKN-1 to promote longevity and collagen synthesis in *C. elegans*. (**a**) The effect of pimozide on *skn-1* mRNA expression in N2 worms. (**b**,**c**) Representative fluorescence micrographs (**b**) and quantitative scoring analysis (**c**) showing the effect of pimozide on SKN-1B/C::GFP expression. (**d**,**e**) The effect of pimozide on mRNA expression of collagen genes (**d**) and hydroxyproline content (**e**) in *skn-1*(*zu67*) mutant worms. (**f**) Survival curves for *skn-1* (*zu67*) mutant worms maintained on NGM plates containing either vehicle (Con, 0.1% DMSO) or pimozide. Replicates are shown in Figure S4. Sample sizes and log-rank *p*-values are summarized in Table S5. Data are mean ± SEM. Asterisks indicate statistical significance in the corresponding indicated pimozide versus Con or *skn-1*(*zu67*) versus N2 (**, *p* < 0.01; ***, *p* < 0.001; ****, *p* < 0.0001). Error bars: SEM. Con: control. DMSO: dimethyl sulfoxide. NGM: nematode growth medium.

SKN-1, the *C. elegans* ortholog of mammalian Nrf2, is a highly conserved transcription factor essential for the oxidative stress response [37]. Notably, SKN-1 maintains tissue homeostasis and delays aging by driving extracellular matrix (ECM) remodeling, with downstream collagens contributing to lifespan extension [38]. Building upon this regulatory axis, we hypothesized that pimozide extends *C. elegans* lifespan via SKN-1 signaling to drive downstream collagen expression. Consistent with this, we found that pimozide treatment significantly upregulated *skn-1* expression and promoted its nuclear localization, providing direct functional evidence of SKN-1 activation (Figure 5a–c). Furthermore, we assessed the efficacy of pimozide in *skn-1*(*zu67*) mutants. As expected, it failed to increase hydroxyproline content, upregulate collagen genes, or extend lifespan in the *skn-1* mutants (Figure 5d–f and Figure S4, Table S5). Together, these findings demonstrate that pimozide promotes the activation of SKN-1, which is required to drive collagen synthesis and extend nematode lifespan.

## 4. Discussion

Although significant progress has been achieved in the research and development of anti-aging therapeutics, most candidate agents remain in the preclinical or early clinical stages, facing challenges related to feasibility, safety, and clinical translation [3]. In contrast, repurposing approved medications offers advantages for geroprotector development, owing to their well-established human safety profiles and pharmacokinetics [3,4]. Among these, psychotropic drugs targeting the monoaminergic system, including antidepressants, psychostimulants, and mood stabilizers, have demonstrated the potential to extend lifespan [7,8,9,10,11]. On one hand, these agents act via classical monoamine-dependent pathways. Given that neurotransmitters such as serotonin (5-HT) and dopamine play key roles in nutrient sensing and healthspan regulation, intervening in these pathways can effectively modulate lifespan [39]. On the other hand, these drugs can also delay aging via monoamine-independent mechanisms. This study focuses on the antipsychotic pimozide, which has not been previously characterized as a geroprotective agent.

This study investigated the antipsychotic agent pimozide and found that at a low concentration of 0.1 μM, it significantly prolonged the lifespan of *C. elegans*, improved healthspan, and enhanced resistance to environmental stress. Clinically, the therapeutic dosage range for pimozide is approximately 1–20 mg/day, with doses exceeding 10 mg frequently inducing significant adverse side effects [40,41]. Our study shows that pimozide exerts lifespan-extending effects in *C. elegans* at relatively low concentrations. However, substantial pharmacokinetic differences exist between *C. elegans* and mammals, and thus our findings remain at the preclinical stage. Nevertheless, this study provides an experimental basis for further pharmacological evaluation and dose optimization in higher model organisms.

Pimozide is pharmacologically defined as a dopamine D2 receptor (D2R) antagonist [13], and its nematode homologs DOP-2 and DOP-3 have been implicated in lifespan regulation [12,42]. However, the role of dopamine in aging appears complex: both activation of DOP-2 and blockade of DOP-3 have been reported to extend lifespan in *C. elegans*, suggesting that dopamine signaling has both positive and negative effects on lifespan depending on the context [12,42]. Although our transcriptomic data revealed no significant changes in *dop-2* or *dop-3* expression upon pimozide treatment (Figure S3d), such transcriptional stability does not preclude receptor antagonism or functional modulation at the protein level. In addition, previous evidence indicates that low pimozide doses (≤0.1 μM) have negligible effects on neuronal dopamine content, whereas higher concentrations are required to inhibit vesicular dopamine uptake and reduce presynaptic dopamine levels [43]. Given that pimozide optimally extended lifespan only at low concentrations, these doses are likely insufficient to disrupt overall dopamine homeostasis. In light of these complexities, the current data do not establish whether the longevity-promoting effect of pimozide depends on dopaminergic signaling. Targeted pharmacological or genetic experiments utilizing *dop-2* and *dop-3* mutants will be required in future work to definitively clarify this point.

To elucidate the mechanisms of pimozide-induced longevity, we used RNA sequencing to screen downstream targets. Transcriptomic profiling revealed that pimozide significantly upregulates a broad array of collagen genes and increases collagen content in *C. elegans*. As a primary structural constituent of the ECM, collagen declines with age, and ECM homeostasis is intimately linked to the aging process [44]. In *C. elegans*, collagens are essential for cuticle formation, which protects the worm from environmental stress and maintains morphology and motility [35]. Beyond these structural roles, cuticular collagens integrate with molecular signaling networks to regulate organismal lifespan [36], positioning ECM remodeling as a potential longevity signal and anti-aging target. Importantly, the efficacy of various longevity interventions depends on collagen. For instance, overexpression of *col-10*, *col-13*, and *col-120* is sufficient to extend nematode lifespan, whereas knockdown of these genes abrogates the longevity effects of multiple longevity interventions [36,38,45]. Notably, pimozide and other longevity interventions (such as dietary restriction) both target and upregulate many of these same collagen genes [36]. Intriguingly, an earlier study in premetamorphic bullfrog larvae also observed that pimozide enhanced collagen synthesis by stimulating the release of endogenous prolactin [46]. This cross-species observation suggests an evolutionary conservation of pimozide’s capacity to promote collagen anabolism.

The observed upregulation of collagen gene expression in our transcriptomic data prompted further investigation of the upstream regulatory networks. The transcription factor SKN-1 (the nematode homolog of mammalian Nrf2) can integrate metabolic and stress-responsive signaling to promote longevity, and has been implicated as a key mediator for various geroprotective interventions [37]. It functions not only as a central hub for antioxidant defense, detoxification, and proteostasis, but also as a key regulator driving ECM remodeling and collagen gene expression [38]. Crucially, the present study confirmed that pimozide activates SKN-1 by driving its nuclear translocation. In addition, the beneficial effects of pimozide, including increased hydroxyproline content, upregulation of collagen genes, and lifespan extension, were markedly attenuated in *skn-1* mutant nematodes, indicating that *skn-1* is essential for pimozide-mediated downstream collagen synthesis and longevity effects. Notably, collagen genes are considered downstream effectors of SKN-1 in various longevity interventions [36], suggesting that their induction may directly contribute to lifespan extension. On the other hand, many stress responses in *C. elegans* trigger systemic cuticle remodeling as a protective adaptation [47], which can indirectly enhance survival. Consequently, future research is required to clarify whether this collagen remodeling is a specific longevity driver or a broader stress response, and whether SKN-1 activation strictly precedes it or acts independently.

Functionally, SKN-1 is highly responsive to diverse upstream stimuli, serving as a primary sensor for ROS and xenobiotic stress [38]. Given that many longevity pharmacological agents act as mild chemical stressors to induce a hormetic response [48], it is highly plausible that low-dose pimozide functions as a xenobiotic trigger. Administration of pimozide at an optimal, low concentration may elicit a mild, beneficial stress signal that promotes SKN-1 activation. Furthermore, SKN-1 does not operate in isolation within the aging regulatory network; it extensively cross-talks with the classical insulin/IGF-1 signaling (IIS) pathway. SKN-1 is a well-established downstream target of the IIS [21]; IIS inhibition can promote SKN-1 nuclear translocation, thereby increasing the expression of antioxidant and ECM-related genes [38]. Moreover, activation of SKN-1 can act synergistically with the IIS pathway to co-activate downstream longevity targets [49].

## 5. Conclusions and Limitations

In summary, this study demonstrates that pimozide extends lifespan and healthspan in an SKN-1-dependent manner, accompanied by enhanced collagen synthesis. However, several limitations should be noted, including reliance on a single effective concentration (0.1 μM), the use of an invertebrate model without established mammalian confirmation, and the absence of individual mutant validation for the upregulated collagen genes to clarify their contribution to longevity. Notwithstanding the above constraints, these findings highlight the significant potential of pimozide as a drug-repurposing candidate for anti-aging interventions, with future work requiring dose optimization, pharmacokinetic profiling, and validation in mammalian models.

## Data Availability

The sequencing data presented in this study are available in GEO at https://ncbi.nlm.nih.gov/geo/query/acc.cgi?acc=GSE338120 (accessed on 19 August 2026). All other data in this study are included in the article and Supplementary Materials.

## Supplementary Materials

The following supporting information can be downloaded at: https://www.mdpi.com/article/10.3390/antiox15091217/s1, Figure S1: Pimozide extends lifespan of *C. elegans*. Survival curves for nematodes maintained on NGM plates containing either vehicle (Con, 0.1% DMSO) or pimozide under 36.5 °C (a,c) or 20 °C (d,e). Sample sizes and log-rank *p*-values are summarized in Table S3. Con, control; DMSO, dimethyl sulfoxide; NGM, nematode growth medium; Figure S2: Pimozide-treated *C. elegans* show enhanced stress resistance. (a,b) The effect of pimozide on OP50 growth (a) and the number of progeny (b); (c–f) Representative survival curves show vehicle- or pimozide-treated worms challenged with UV irradiation at 120 mJ/cm^2^ (c,d) or 500 μM juglone (e,f) until all the worms died. Sample sizes and log-rank *p*-values are summarized in Table S4. Con, control; UV, ultraviolet; Figure S3: Transcriptomic profiling of pimozide-treated *C. elegans*. (a) PCA score plot; (b) KEGG pathway enrichment analysis; (c) GO enrichment analysis of downregulated DEGs; (d) FPKM values of *dop-2* and *dop-3*. Con, control; DEGs, differentially expressed genes; Figure S4: Pimozide extends lifespan in a *skn-1*-dependent manner. Survival curves for *skn-1* (*zu67*) mutant nematodes maintained on NGM plates containing either vehicle (Con, 0.1% DMSO) or pimozide. Sample sizes and log-rank *p*-values are summarized in Table S5. Con, control; DMSO, dimethyl sulfoxide; NGM, nematode growth medium; Table S1: List of primers used to quantify mRNA; Table S2: Summary of RNA-sequencing quality-control and read-alignment metrics for each sample. Table S3: Lifespan assay of pimozide-treated *C. elegans*. Related to Figure 1 and Figure S1; Table S4: Lifespan assay of pimozide-treated *C. elegans* under stress conditions. Related to Figure 3a,b and Figure S2c–f; Table S5: Lifespan assay of *skn-1* mutant strains treated with pimozide. Related to Figure 5f and Figure S4.
