# Supplementary material for: Repurposing Pimozide as a Geroprotector: Lifespan Extension via SKN-1 Activation and Collagen Remodeling in Caenorhabditis elegans
Source: Antioxidants (Basel). 2026 Sep 21;15(9):1217. doi: 10.3390/antiox15091217 (PMC13603762; doi:10.3390/antiox15091217)
Supplement: Supplementary file 1 [file antioxidants-15-01217-s001.zip › Supplementary table.pdf]

Table S1. List of primers used to quantify mRNA.

Symbol		Sequence（5'-3'）
act-1	For	TCGGTATGGGACAGAAGGAC
	Rev	CATCCCAGTTGGTGACGATA
col-3	For	CAAGGGACCATCTGGACCAAACG
	Rev	GTCCTGGTGCTCCTGGTGTAATTG
col-144	For	TGCCAATGCGAGCCAACTAAGC
	Rev	AGGTGAGTGGAGCGAAGGTAGC
col-167	For	CCGCACTCAAGTTCTCGCCAAG
	Rev	CAGATACTCCACATCCGCAGCATC
col-41	For	ACAGCCAGGAGAGCCAGGATG
	Rev	GCGTCGTGACCAGCGTTTCC
col-54	For	CCTTCAAGCACTACTCCGATGTCG
	Rev	GGGCAGCCACATGACTTATCTTCG
col-128	For	AGGTGTGCCAGGAGCGAAGG
	Rev	CCAATCCAGATGGTCCAGGTTGA
col-131	For	TCCTTTCGGTGCTGGTATTTGTCC
	Rev	CCACGATGCTTCACGGAGAACC
col-176	For	GACCACCAGGACCACCAGGAC
	Rev	TGGGCAGTTGATTGGAGCAAAGG
Skn-1	For	GTTCCCAACATCCAACTACG
	Rev	TGGAGTCTGACCAGTGGATT

Table S2. Summary of RNA-sequencing quality-control and read-alignment metrics for each sample.

Sample	Raw reads	Valid Data	Valid ratio (%)	Q30 (%)	GC content (%)	Mapping rate (%)
CONTROL 1	49376792	47979738	97.17	98.06	46.0	97.63
CONTROL 2	41517804	40376678	97.25	97.84	45.5	97.59
CONTROL 3	36402514	35445978	97.37	97.96	46.0	97.51
CONTROL 4	42200108	41191558	97.61	97.98	46.0	97.59
PIMOZIDE 1	37250794	36238424	97.28	98.06	46.0	97.67
PIMOZIDE 2	42100952	40972848	97.32	97.96	46.0	97.66
PIMOZIDE 3	41723474	40438272	96.92	97.97	46.0	97.49
PIMOZIDE 4	40791720	39681182	97.28	98.03	46.0	97.39

Table S3. Lifespan assay of pimozide-treated *C. elegans*.

Strain	Experiment	Condition	Mean	Median	Max	Hazard ratio (HR)	95% confidence interval (CI)	Dead/censored worms	*p* value (survival)	% change	Figure on text
N2 (36.5℃)	1	Con	6.5	7.0	9.6			50			Fig. 1a
		0.1μM	8.8	9.0	13.5	0.39	0.26-0.59	49	<0.0001	35.4	
		0.2μM	7.7	8.0	11.2	0.62	0.42-0.92	50	0.0042	18.5	
		0.5μM	6.9	7.0	10.4	0.81	0.54-1.19	50	0.1965	6.2	
	2	Con	8.0	8.0	11.2			50			Fig. S1b
		0.1μM	10.0	10.0	14.0	0.44	0.30-0.67	49	<0.0001	25.0	
		0.2μM	9.5	9.0	13.0	0.54	0.36-0.81	48	0.0008	18.8	
		0.5μM	9.3	9.0	12.8	0.58	0.39-0.86	50	0.0019	16.3	
	3	Con	8.2	8.0	12.0			50			Fig. S1c
		0.1μM	10.0	10.0	14.2	0.51	0.34-0.76	50	<0.0001	22.0	
		0.2μM	9.5	9.0	13.4	0.62	0.41-0.91	50	0.0043	15.9	
		0.5μM	8.2	8.0	12.8	0.91	0.62-1.35	50	0.6633	0	
N2 (20℃)	1	Con	18.3	18.0	23.6			50			Fig. 1b
		0.1μM	26.4	27.0	33.0	0.12	0.07-0.22	50	<0.0001	44.3	
	2	Con	19.6	20.0	25.8			50			Fig. S1d
		0.1μM	24.1	24.0	30.6	0.37	0.24-0.57	50	<0.0001	23.0	
	3	Con	19.5	20.0	25.4			50			Fig. S1e
		0.1μM	25.0	26.0	31.4	0.25	0.15-0.41	50	<0.0001	28.2	

Table S4. Lifespan assay of pimozide-treated *C. elegans* under stress conditions.

Stress	Experiment	Condition	Mean	Median	Max	Hazard ratio (HR)	95% confidence interval (CI)	Dead/censored worms	*p* value (survival)	% change	Figure on text
UV (120 mJ/cm^2, 5min)	1	Con	3.1	3.0	4.3			50			Fig. 3a
		Pimozide	3.6	3.5	5.2	0.59	0.39-0.90	50	0.0023	16.1	
	2	Con	3.4	3.5	4.5			49			Fig. S2c
		Pimozide	4.2	4.0	5.8	0.44	0.28-0.69	50	<0.0001	23.5	
	3	Con	3.4	3.5	4.5			49			Fig. S2d
		Pimozide	4.5	4.5	6.2	0.32	0.20-0.52	50	<0.0001	32.4	
Juglone (500 μM)	1	Con	7.5	8.0	10.6			49			Fig. 3b
		Pimozide	10.5	10.0	15.4	0.31	0.19-0.49	50	<0.0001	40.0	
	2	Con	7.8	8.0	11.4			50			Fig. S2e
		Pimozide	9.0	9.0	13.0	0.63	0.42-0.95	50	0.0094	15.4	
	3	Con	6.9	7.0	10.4			50			Fig. S2f
		Pimozide	10.4	10.0	14.8	0.28	0.18-0.45	50	<0.0001	50.7	

Worms were sorted onto NGM plates and exposed to different stress conditions for lifespan assessment. *p* value for Kaplan-Meier survival analysis was calculated with the log-rank method and that for survival analysis calculated with the Mantel-Cox method. Maximum lifespan was defined as the lifespan of the last 10% of the population. Percentage change based on mean lifespan. Hazard ratios (HRs) and their 95% CIs were calculated using the Cox proportional hazards model.

Table S5. Lifespan assay of mutant strains treated with pimozide.

Mutant strain	Experiment	Condition	Mean	Median	Max	Hazard ratio (HR)	95% confidence interval (CI)	Dead/censored worms	*P* value (survival)	Figure on text
skn-1 (*zu67*)	1	Con	18.8	19.0	23.0			51		Fig. 5f
		Pimozide	18.4	18.0	24.2	1.01	0.68-1.51	50	0.9579	
	2	Con	17.9	18.0	23.8			50		Fig. S4a
		Pimozide	19.1	19.0	24.8	0.72	0.49-1.07	52	0.0659	
	3	Con	19.3	19.0	24.4			50		Fig. S4b
		Pimozide	18.6	19.0	24.8	1.10	0.75-1.63	52	0.5817	

Worms were sorted onto NGM plates and assessed for lifespan. *p* value for Kaplan-Meier survival analysis was calculated with the log-rank method and that for survival analysis calculated with the Mantel-Cox method. Maximum lifespan was defined as the lifespan of the last 10% of the population. Hazard ratios (HRs) and their 95% CIs were calculated using the Cox proportional hazards model.
